# Microbiome and metabolite signatures for cirrhosis to HCC risk stratification: progress, controversies, and gaps

**DOI:** 10.3389/fcimb.2026.1793213

**Published:** 2026-03-16

**Authors:** Yanan Duan, Mengting Yang, Miaomiao Li, Yu Sun, Shiguo Liu

**Affiliations:** Department of Medical Genetics, Affiliated Hospital of Qingdao University, Qingdao, Shandong, China

**Keywords:** biomarker, gut microbiota, gut-liver axis, hepatocellular carcinoma, liver cirrhosis

## Abstract

The progression from cirrhosis to hepatocellular carcinoma (HCC) is a key outcome in the management of chronic liver disease. This process has a long incubation period and significant individual differences, making early warning still difficult. Clinical follow-up mainly relies on imaging examinations and alpha fetoprotein, but the ability to identify high risk precancerous states is limited. The imbalance of gut microbiota and its metabolites may occur earlier than the visible stage of tumors. They can affect barrier integrity, chronic inflammation, immune surveillance, and metabolic homeostasis through the gut liver axis, and participate in the formation of a pro tumor microenvironment. Therefore, such changes may provide more upstream risk stratification clues for the population with cirrhosis. This article summarizes previous research evidence and summarizes the common microbiome and metabolite characteristics of cirrhosis and high-risk populations, including a decrease in short chain fatty acid (SCFA) related symbiotic bacteria, an increase in inflammation related bacteria, bile acid spectrum shift, and other intestinal derived metabolite abnormalities. This article also outlines the key mechanisms that these features may correspond to, such as barrier damage and microbial translocation, immune suppression, etc. There are still significant uncertainties at present. The effect of SCFA is context dependent. Different etiologies, diets, medications, and complications can lead to significant confounding and affect cross cohort consistency. Subsequent research requires longitudinal cohort validation and the promotion of multi omics integration and the construction of interpretable predictive models to support clinical translation.

## 1 Introduction

Cirrhosis and hepatocellular carcinoma (HCC) are one of the leading causes of chronic liver disease-related deaths worldwide (Liu et al., 2022; Stockdale et al., 2025). Cirrhosis represents the deep remodeling of liver structure and function, and is also the underlying pathological state of most HCC occurrences (Johnson et al., 2022; Winans et al., 2023; Stockdale et al., 2025). Clinical follow-up mainly relies on imaging examinations and serum alpha fetoprotein monitoring, but these methods still have limited recognition of high-risk precancerous states, making it difficult to capture the dynamic changes of carcinogenic mechanisms during the course of the disease and provide sensitive warning signals (Huang et al., 2023; Lani et al., 2024; Hlady et al., 2025; Lyu et al., 2025).

Therefore, barrier disruption and microbial translocation may represent in liver disease, fewer have specifically focused on how stage-associated microbial and metabolic alterations may refine risk stratification during the transition from cirrhosis to HCC (Li et al., 2025; Ma et al., 2025). Under physiological conditions, the liver continuously receives intestinal derived nutrients, microbial metabolites, and microbial associated molecular patterns (MAMPs) through the portal vein. At the same time, bile acids secreted by the liver and various immune regulatory molecules continue to act on the intestine, thereby affecting the composition of the microbiota and barrier function. This bidirectional coupling relationship constitutes the gut-liver axis (Beyoğlu and Idle, 2022; Guan et al., 2022; Scarpellini et al., 2024). When chronic liver disease progresses to cirrhosis, this balance is often disrupted (Chen et al., 2020; Hsu and Schnabl, 2023; Singh et al., 2023). The combination of factors such as reduced bile secretion, intestinal motility disorders, portal hypertension, and decreased local immune defense can damage the barrier and increase the sustained translocation of microorganisms and their products to the liver, thereby causing the liver to enter a low but sustained state of immune stimulation (Gedgaudas et al., 2022; Zeng et al., 2025). Therefore, barrier disruption and microbial translocation may represent permissive conditions that facilitate a pro-inflammatory and pro-tumor microenvironment, although direct causal relationships remain to be fully established (Liu et al., 2022; Yin et al., 2025).

Unlike previous mechanism-oriented reviews, this article specifically reframes gut microbiota and metabolite alterations as candidate transitional signals for risk stratification during the cirrhosis to HCC window. Rather than summarizing individual pathways in isolation, we integrate microbial, metabolic, and immune features into stage oriented framework, highlight cross cohort inconsistencies and context dependency, and discuss their implications for constructing interpretable predictive models.

## 2 Gut microbiota and gut-liver axis

The gut microbiota is a complex microbial ecosystem that colonizes the gastrointestinal tract and is one of the largest and most diverse symbiotic microbial communities in the human body, roughly equivalent in number to host cells (Sender et al., 2016; Gupta et al., 2019). Under physiological conditions, microbial communities participate in nutrient metabolism, mucosal barrier maintenance, immune regulation, and produce various bioactive metabolites. Due to the continuous reception of intestinal derived nutrients, metabolites, and microbial signals through the portal vein, the stability of intestinal ecology is closely related to liver metabolism and immune environment, and is therefore considered an important upstream factor in the occurrence and progression of chronic liver disease (Liu et al., 2016; Allam-Ndoul et al., 2020; Zhu et al., 2020).

The gut-liver axis describes the bidirectional anatomical and physiological connection between the gut and liver. The portal vein system and biliary system allow gut derived nutrients, microbial metabolites, and MAMPs to directly enter the liver, avoiding systemic circulation dilution (Ridlon et al., 2010; Kakiyama et al., 2013; Ridlon et al., 2016; Yang and Duan, 2016; Milosevic et al., 2019). On the contrary, the liver secretes molecules such as bile acids and antimicrobial peptides, which continuously affect the composition of the microbiota, mucosal immunity, and barrier integrity, thereby forming a tightly coupled bidirectional regulatory network.

Bile acids are also signaling molecules that can regulate lipid metabolism, glucose homeostasis, and inflammatory response through pathways such as FXR-FGF19. The microbiota can also feedback on CYP7A1 expression and bile acid pool size by altering bile acid composition and receptor activation status (Sayin et al., 2013; Tripathi et al., 2018). Therefore, bile acid microbiota receptor signaling constitutes the intersection of metabolism and immunity in the gut-liver axis.

The integrity of the intestinal barrier determines the strength of intestinal signals entering the portal vein. Under physiological conditions, a single layer of epithelial cells, along with tight junctions, mucus layer, antimicrobial peptides, and immunoglobulin A (IgA), form a multi-layered composite barrier that restricts the entry of bacteria and their products into the bloodstream (Hardy et al., 2013; Peterson and Artis, 2014; Luissint et al., 2016). When inflammation persists or the microbiota becomes imbalanced, the barrier is gradually damaged, and bacteria, endotoxins, and other microbial products are more likely to cross the epithelium and enter the liver through the portal vein, triggering Kupffer cell activation and the release of pro-inflammatory factors such as TNF-α and IL-6, thereby exacerbating inflammation and promoting fibrosis (Odenwald and Turner, 2017; Allaire et al., 2018). Therefore, the barrier state not only affects the intensity of intestinal input, but also participates in the transition of chronic liver disease from relative homeostasis to sustained imbalance.

Short chain fatty acids (SCFAs) include acetic acid, propionic acid, and butyric acid, mainly produced by fermentation of dietary fiber (Peng et al., 2009; Macfarlane and Macfarlane, 2012; Jung et al., 2015). SCFAs can also regulate immune cell differentiation through receptors such as GPR41, GPR43, and GPR109A, promote regulatory T cell generation, and inhibit inflammatory responses, indirectly reducing the burden of liver inflammation (Smith et al., 2013; Singh et al., 2014). Relatively speaking, lipopolysaccharides (LPS) are a typical pro-inflammatory signal in the gut-liver axis. Low levels of LPS can maintain the basal activity of Kupffer cells through TLR4, but when the barrier is damaged or the microbiota is dysregulated, leading to an increase in LPS translocation, intrahepatic inflammation is amplified and the progression of liver injury is accelerated (Cornell, 1985; Rao et al., 2004). Meanwhile, some indole derivatives can also regulate mucosal immunity through the AhR or PXR pathways and indirectly exert protective effects on the liver (Bansal et al., 2010; Venkatesh et al., 2014; Lamas et al., 2016; Wlodarska et al., 2017).

In health, bile acid circulation, barrier integrity, and microbial metabolism maintain gut-liver axis homeostasis, supporting immune tolerance and metabolic stability. With progression to cirrhosis, this balance becomes disrupted. Barrier impairment increases translocation of microbial products, contributing to persistent low grade hepatic inflammation. Concurrently, microbial metabolic remodeling leads to reduced protective metabolites and relative accumulation of pro-inflammatory or tumor-associated metabolites. Thus, gut-liver axis imbalance in cirrhosis may not only reflect disease severity but also contribute to a microenvironment permissive for hepatocarcinogenesis.

## 3 Changes in gut microbiota and metabolic profile during the stage of liver cirrhosis

Across sequencing platforms and metabolomic approaches, a relatively reproducible pattern of reduced microbial diversity and functional output has been observed in cirrhosis, suggesting that certain microbial shifts may represent stage associated rather than incidental alterations (Hsu and Schnabl, 2023; Cai et al., 2024; Buttler et al., 2025). Systematic reviews and meta-analyses (Li et al., 2021; Cai et al., 2024) suggest a decline in various functional microbiota associated with SCFAs production, barrier maintenance, and immune homeostasis, such as Ruminococcaceae, Lachnospiraceae, Faecalibacterium, and Coprococcus. Relatively speaking, bacterial communities associated with increased inflammation or opportunistic infections are more common, including Escherichia coli, Enterococcus, Streptococcus, Weissella, and members of the Clostridium family (Loomba et al., 2021; Zeng et al., 2024).

It is worth noting that imbalance is not limited to the bacterial level. Fungal diversity decreases in patients with cirrhosis, and enrichment of Candida albicans, Mucor, and Hansenula is observed in advanced liver disease (Hartmann et al., 2021; Demir et al., 2022; Zeng and Schnabl, 2024). At the same time, the viral community can also undergo remodeling, resulting in a decrease in overall bacteriophage diversity and a decrease in the abundance of certain specific bacteriophages (Lang et al., 2020). Macrogenomics research suggests that during the process from simple fatty liver to significant fibrosis and then to cirrhosis, the abundance of microbiota undergoes continuous changes, with Escherichia coli gradually increasing, while the microbiota related to anti-inflammatory or metabolic regulation continues to decrease (Loomba et al., 2017; Oh et al., 2020). Small intestinal bacterial overgrowth (SIBO) is more frequently observed in cirrhosis. However, interpretation of microbial shifts requires caution due to potential reverse causality and indication bias. Patients with more advanced liver dysfunction are more likely to receive antibiotics, proton pump inhibitors, or lactulose, all of which independently reshape microbial composition and metabolite profiles (Gkolfakis et al., 2023). Therefore, part of the observed enrichment of Enterococcus, Escherichia coli, or altered bile acid and SCFA patterns may reflect treatment exposure or disease severity rather than intrinsic carcinogenic mechanisms.

The reshaping of microbial community structure is usually accompanied by systematic adjustments in metabolic profiles. It should be noted that reported microbial and metabolite alterations derive from heterogeneous biological matrices, including fecal samples, peripheral serum, portal vein blood, and bile. These compartments differ in biological meaning, temporal stability, and susceptibility to systemic influences. For example, fecal microbiota primarily reflect luminal composition, whereas serum or portal vein metabolites may capture host microbe co-metabolism and hepatic clearance capacity. Therefore, direct comparison across sample types requires caution, and analytical stability may vary depending on the specimen source. Secondary non conjugated bile acids such as deoxycholic acid and lithocholic acid are more common in patients with advanced liver cirrhosis (Park et al., 2021; Spatz et al., 2021), suggesting that their levels may be related to the severity of the disease. At the same time, SCFA related changes are also consistent, with decreased production capacity, imbalanced circulation distribution, and gradual accumulation of protein fermentation related metabolites (Awoniyi et al., 2023). Importantly, it remains uncertain whether these microbial alterations actively contribute to tumorigenesis or primarily reflect worsening hepatic function and portal hypertension. For instance, the expansion of Enterococcus or Escherichia coli may result from impaired bile secretion, reduced intestinal motility, or frequent antibiotic exposure in advanced cirrhosis, rather than representing independent carcinogenic drivers. Therefore, distinguishing microbial markers of disease severity from mechanistic mediators of carcinogenesis requires longitudinal and functionally validated studies. In addition, elevated levels of branched chain fatty acids (BCFAs) are often considered a hallmark of enhanced protein fermentation and may be associated with disease severity and metabolic stress (Kawasoe et al., 2022; Yang et al., 2025). Another study (Scharf et al., 2025) suggested that the decrease in SCFA may be accompanied by a decrease in HIF-1 α signaling activity and a decrease in tight junction protein expression, suggesting that metabolic dysfunction may be synchronized with impaired barrier maintenance ability.

The gut-liver axis imbalance in some high-risk patients is characterized by multiple abnormalities occurring simultaneously, often accompanied by persistent inflammatory background, decreased immune regulatory ability, and easier deviation from metabolic homeostasis, and is not completely equivalent to the common pattern of simple cirrhosis (**Table 1**). It should be emphasized that these signals are closer to candidate transitional features and do not necessarily mean that cancer has already occurred. In patients with established HCC on the background of cirrhosis, several studies have reported enrichment of Escherichia coli, Enterococcus, Streptococcus, and Veillonella compared with cirrhosis without HCC. By contrast, in cirrhotic populations without radiologically detectable tumors but considered clinically high risk, similar trends have been observed in some cohorts. However, the temporal relationship and predictive specificity remain uncertain. These overlapping patterns raise the possibility that certain inflammatory associated communities expand progressively along the cirrhosis HCC spectrum, although current cross-sectional data do not allow clear stage discrimination. At the metabolic level, changes in bile acid profiles are more prominent. Elevated deoxycholic acid levels have been consistently reported in cirrhosis associated HCC (Mangan and Latz, 2014; Schnabl et al., 2025). However, whether similar elevations can prospectively distinguish high risk cirrhosis prior to tumor detection remains unclear. In addition, gut derived endogenous metabolites such as ethanol, trimethylamine oxide (TMAO), and phenylacetic acid are more commonly elevated in patients with cirrhosis and HCC, and these changes often occur simultaneously with increased lipid deposition, worsening oxidative stress, and fibrosis progression (Flores-Guerrero et al., 2021; Gan et al., 2023; Nagel et al., 2024). In contrast, the changes in SCFA are more context dependent.

**Table 1:** Gut microbiota and microbial metabolite alterations in cirrhosis associated with hepatocellular carcinoma risk.

Category	Microecological factor	Direction of change in cirrhosis	Main function/biological relevance	Evidence linking to HCC transition	Potential application	References
Short-chain fatty acid-producing bacteria	Faecalibacterium prausnitzii	Reduced abundance	Butyrate production, maintenance of intestinal barrier, anti-inflammatory effects	Persistent depletion associated with cirrhosis progression and increased HCC risk	Risk stratification, probiotic or synbiotic target	(Li et al., 2021; Su et al., 2024)
	Ruminococcaceae	Reduced abundance	Short-chain fatty acid synthesis, immune homeostasis	Marked reduction in pre-HCC stage	Predictive biomarker	(Xing et al., 2025; Zhang et al., 2025)
	Lachnospiraceae	Reduced abundance	Epithelial energy supply, barrier maintenance	Depletion associated with inflammatory amplification and precancerous niche formation	Interventional target	(Petrick et al., 2023; Xing et al., 2025)
Inflammation-associated bacteria	Enterococcus	Increased abundance	Lipopolysaccharide and lipoteichoic acid production, Toll-like receptor activation	Enriched in cirrhosis patients at high HCC risk	Prediction and suppression target	(Shayegan et al., 2021; Cheng et al., 2022)
	Streptococcus	Increased abundance	Oral-derived microbiota, pro-inflammatory activity	Associated with cirrhosis complications and HCC	Predictive marker	(Li et al., 2021; Su et al., 2024)
	Escherichia coli	Increased abundance	Lipopolysaccharide and ethanol production	Stepwise enrichment with fibrosis progression and HCC risk	Risk assessment	(Li et al., 2021; Su et al., 2024)
SIBO-associated bacteria	Staphylococcus aureus	Increased abundance	Endotoxin production, barrier disruption	SIBO linked to cirrhosis decompensation and HCC risk	Interventional window	(Gehrke et al., 2024)
Mycobiome	Candida albicans	Increased abundance	Beta-glucan production, immune activation	Elevated anti-Candida IgG associated with HCC occurrence	Immune-based prediction	(Kim et al., 2022; Song et al., 2023)
	Saccharomyces cerevisiae	Reduced abundance	Commensal fungus, barrier support	Depletion associated with disease severity	Homeostasis marker	(Kim et al., 2022)
Virome	Lactococcus phages	Reduced abundance	Maintenance of bacterial community stability	Phage loss contributes to microbial dysregulation	Ecological stability indicator	(Sasamori et al., 2024)
Bile acids	Deoxycholic acid	Increased level	DNA damage, senescence-associated secretory phenotype activation	Directly promotes HCC and suppresses natural killer T cell function	High-risk metabolic marker	(Su et al., 2024; Zeng et al., 2024)
	Lithocholic acid	Increased level	Hydrophobic toxicity	Associated with fibrosis and tumor risk	Predictive marker	(Qu et al., 2022; Sha et al., 2025)
Short-chain fatty acids	Acetate	Context-dependent alteration	Barrier protection or tumor metabolic substrate	Dual biological effects	Requires stratified interpretation	(Han et al., 2023; Pan et al., 2025)
	Butyrate	Reduced level	Histone deacetylase inhibition, anti-inflammatory effects	Depletion weakens antitumor barrier	Interventional target	(Adorini and Trauner, 2023; Shi et al., 2023)
Other metabolites	Endogenous ethanol	Increased level	Oxidative stress, DNA damage	Ethanol-producing bacteria promote HCC	Targeted eradication	(Zeng and Schnabl, 2024)
	Trimethylamine N-oxide	Increased level	Metabolic inflammation and fibrosis	Associated with liver disease progression and HCC risk	Risk marker	(Loomba et al., 2017; Oh et al., 2020)
Functional pathways	Lipopolysaccharide biosynthesis	Enhanced activity	Toll-like receptor 4 activation	Promotes chronic inflammation	Mechanistic indicator	(Chen et al., 2023; Golonka et al., 2024)
	Seven-alpha dehydroxylation	Enhanced activity	Secondary bile acid generation	Facilitates immune escape	Metabolic target	(Muthiah et al., 2022; Zeng et al., 2024)

## 4 Potential mechanisms linking gut microbiota and metabolite imbalance to the transition from cirrhosis to hepatocellular carcinoma

**Figure 1:**
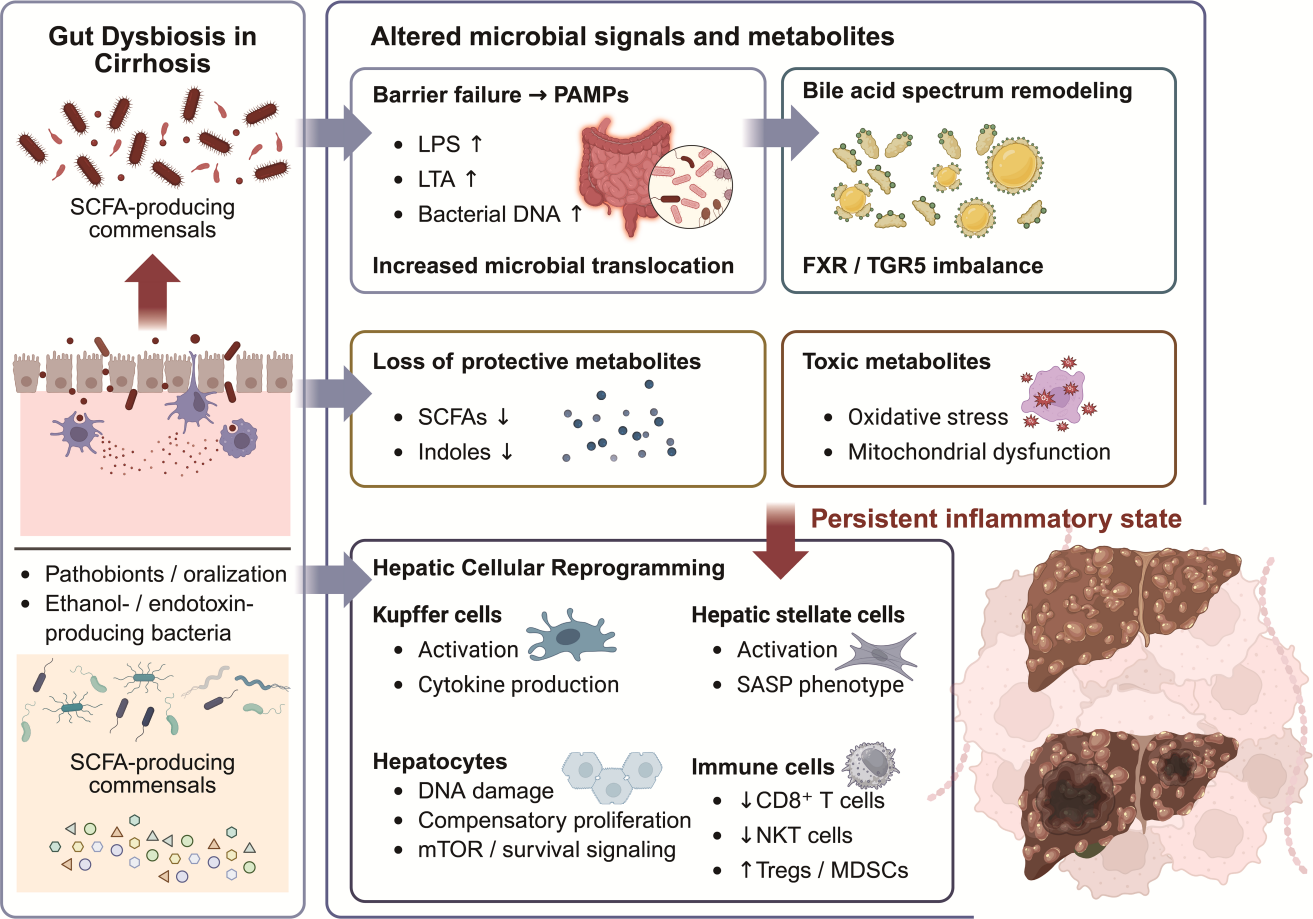
Microbiota-driven mechanisms promoting the transition from liver cirrhosis to hepatocellular carcinoma. Gut dysbiosis in cirrhosis represents a critical upstream event that initiates and sustains multiple pro-tumorigenic processes along the gut-liver axis. Cirrhosis-associated microbial alterations are characterized by a reduction in SCFA-producing commensals and an expansion of pathobionts, including oralized taxa and ethanol- or endotoxin-producing bacteria. These changes contribute to intestinal barrier failure, leading to increased translocation of PAMPs, such as LPS, LTA, and bacterial DNA, from the gut to the liver. Concurrently, dysbiosis-driven remodeling of the bile acid spectrum results in an accumulation of secondary bile acids, accompanied by imbalanced signaling through bile acid receptors FXR and TGR5. In parallel, the loss of protective microbial metabolites, including SCFAs and indole derivatives, weakens epithelial barrier integrity and immunoregulatory pathways, while the accumulation of toxic microbial metabolites promotes oxidative stress and mitochondrial dysfunction. Together, these altered microbial signals establish a persistent low-grade inflammatory state that acts as a permissive background rather than a discrete pathway. Sustained inflammatory and metabolic stress drives hepatic cellular reprogramming across multiple cell types. Kupffer cells and hepatic stellate cells undergo chronic activation, with stellate cells acquiring a senescence-associated secretory phenotype. Hepatocytes experience ongoing DNA damage, compensatory proliferation, and activation of pro-survival signaling pathways. At the same time, the hepatic immune landscape shifts toward immunosuppression, characterized by reduced CD8^+^ T cells and NKT cells and expansion of regulatory T cells and myeloid-derived suppressor cells. Collectively, these processes reshape the hepatic microenvironment into a pro-tumorigenic niche that facilitates the progression from cirrhosis to hepatocellular carcinoma. Potential intervention points targeting the gut microbiota, microbial metabolites, barrier integrity, bile acid signaling, and immune regulation are indicated.

The transition from cirrhosis to HCC represents a biologically unstable window in which immune tolerance, inflammatory thresholds, and metabolic selection pressures are simultaneously redefined. In this context, gut derived signals may function not merely as bystanders but as modulators that lower the threshold for malignant transformation. The gut microbiota and its metabolites provide continuous gut derived signals that may influence inflammatory tone, immune selection pressure, and metabolic substrate utilization in the liver. The evolution from cirrhosis to HCC is more likely to be a gradual process of gut liver axis imbalance rather than a single carcinogenic event. Environment towards a pro tumor state (**Figure 1**).

### 4.1 Intestinal barrier injury

In the stage of cirrhosis, there is often a persistent imbalance in the intestinal liver axis homeostasis. This process is usually driven by a combination of multiple factors, including reduced bile secretion, weakened intestinal motility, portal hypertension, and decreased local immune defense (Guan et al., 2022; Kronsten et al., 2022; Tilg et al., 2022; Zeng et al., 2025). Subsequently, bacteria and their products are more likely to cross barriers and enter the liver through the portal vein (Assimakopoulos et al., 2024; Smets et al., 2025; Xing et al., 2025). Barrier damage itself is not equivalent to a carcinogenic event, but it can reshape the underlying immune background in which the liver operates, shifting the liver from relative immune tolerance to a long-term, low-level but sustained immune stimulation state (Chen et al., 2020; Petrick et al., 2023; Zhang et al., 2025). Under constant doses of carcinogens, animal models have shown that simply breaking the intestinal barrier can accelerate the occurrence of hepatocellular carcinoma (Liu et al., 2022; Gehrke et al., 2024; Yin et al., 2025), suggesting that barrier damage may modulate susceptibility to carcinogenic processes rather than acting as an independent initiating factor.

### 4.2 Chronic inflammatory signals driven by microbial products

When gut derived microorganisms and their products continue to enter the liver, Kupffer cells, hepatic stellate cells, sinusoidal endothelial cells, and liver cells can all respond through pattern recognition receptors (Chen et al., 2021; Kim et al., 2022). LPS induces sustained production of pro-inflammatory cytokines such as TNF - α and IL-6 through TLR4, maintaining a low degree of inflammation in the liver (Chen et al., 2021; Qu et al., 2022; Sasamori et al., 2024; Sha et al., 2025). In addition to LPS, Gram positive bacteria associated lipoteichoic acid can activate the inflammatory pathway through TLR2, and peptidoglycan and other components can amplify the inflammatory response through NOD2 (Getachew et al., 2021; Zhou et al., 2021; Omaru et al., 2022). Under long-term stimulation, the liver is more likely to enter a state of repeated repair and continuous fibrosis progression. Hepatic stellate cells are sensitive to PAMP stimulation, and continuous stimulation can promote age-related secretory phenotypes, as well as long-term release of pro-inflammatory cytokines, chemokines, and matrix remodeling molecules (Shayegan et al., 2021; Cheng et al., 2022; Liu et al., 2022). This combination is closer to the superposition of sustained selection pressure and repair pressure, which is a common pathological background for the gradual establishment of tumor related microenvironment.

### 4.3 Immune suppression and immune surveillance function impaired

The occurrence of liver cirrhosis related HCC not only depends on the accumulation of mutations, but also on the gradual decline of immune clearance ability (Lurje et al., 2021; Kim and Seki, 2025). In the context of cirrhosis, the effector function of CD8 ^+^ T cells is limited, the recruitment of natural killer T cells in the liver is reduced, and the proportion of regulatory T cells and myeloid derived inhibitory cells is increased, resulting in a systemic bias in immune composition and function (Lurje et al., 2021; Chivite-Lacaba et al., 2025; Liu et al., 2025). More importantly, the time window. Some studies (Shriki et al., 2021) suggest that this immunosuppressive feature may appear when tumors have not yet been identified by imaging. However, most of the available data are cross-sectional, and although immunosuppressive features have been observed in cirrhotic patients without radiologically detectable tumors, longitudinal evidence confirming their temporal precedence over tumor initiation remains limited. After the immune clearance threshold is upregulated, abnormal cells are more likely to escape. At the same time, the increased regenerative pressure during the cirrhosis stage leads to more frequent cell replication, an increased chance of mutation occurrence, and a decreased probability of clearance. The combination of the two is more conducive to fixing the pre cancerous niche.

### 4.4 Systemic carcinogenic effects of microbial metabolites

Microbial metabolites are regulatory factors that shape the immune and metabolic microenvironment during the transition from cirrhosis to HCC. Bile acids and SCFAs are the two most commonly discussed metabolites (Song et al., 2023; Pan et al., 2025). Indole tryptophan metabolites are also closely related to barriers and immune regulation (Mrdjen et al., 2023; Jia et al., 2024; Luo et al., 2025).

When bile secretion decreases and microbiota remodeling occurs in parallel during the stage of cirrhosis, the composition of bile acids is more prone to shift, and the proportion of secondary bile acids increases, especially deoxycholic acid (DCA) increases (Muthiah et al., 2022; Adorini and Trauner, 2023; Chen et al., 2023; Han et al., 2023; Shi et al., 2023; Golonka et al., 2024). Experimental studies indicate that DCA can induce DNA damage and activate pro-inflammatory and anti-apoptotic pathways (Cong et al., 2024; Morgan et al., 2025). However, whether these mechanisms directly translate into human carcinogenesis requires further validation. Secondary bile acids may also weaken anti-tumor immunity. Previous studies (Sun et al., 2021; Cheng et al., 2023) suggest that it can affect CXCR6 natural killer T cell recruitment by regulating CXCL16 expression, thereby reducing local immune surveillance.

The role of SCFAs in the metabolic environment of cirrhosis and HCC is not fixed (Liu et al., 2021; Dicks, 2024; Mann et al., 2024). Acetate can be utilized by tumor cells under conditions of metabolic stress or hypoxia as a carbon source for processes such as lipid synthesis or protein glycosylation, thereby supporting their growth needs (Zhu et al., 2022; He et al., 2023; Zhou et al., 2023; Esquea et al., 2024; Wang et al., 2024). SCFAs have been shown to regulate immune cell differentiation and function in experimental settings, although their net effect in cirrhosis-associated carcinogenesis appears context dependent (Che et al., 2023; Ma et al., 2023; Lee et al., 2025). This phenomenon suggests that SCFAs are more like regulatory signals that depend on concentration, tissue distribution, and host immune metabolism status, rather than fixed directional protective factors (Zhao et al., 2021; Beckman et al., 2025; Zhang et al., 2025).

Indole tryptophan metabolites are closely related to barrier repair and immune regulation (Mrdjen et al., 2023; Jia et al., 2024; Luo et al., 2025). Under normal circumstances, they can promote IL-22 production through AhR, thereby supporting epithelial repair and enhancing mucosal defense (He et al., 2023; Zhang et al., 2023; Zhou et al., 2024). In the stage of cirrhosis, the ability to produce protective indole metabolites often decreases. After the AhR-IL-22 axis weakens, the barrier repair ability decreases, and microbial products are more likely to cross the barrier and enter the body (Mrdjen et al., 2023; Wang et al., 2023; Jia et al., 2024; Luo et al., 2025). The AhR signal can also affect the functional status of liver immune cells, and its imbalance may be more conducive to the progression of tumor related processes under specific conditions (Yu et al., 2022; Holloman et al., 2023; Lv et al., 2024; Su et al., 2024).

In summary, microbial metabolites in the cirrhosis HCC spectrum appear to converge on immune modulation, inflammatory amplification, and metabolic reprogramming. Their effects are stage dependent and context sensitive, suggesting that metabolite signatures represent coordinated shifts in host microbe equilibrium rather than isolated oncogenic triggers.

## 5 Conclusion and Outlook

This review proposes that microbiome and metabolite alterations during cirrhosis should not be viewed solely as downstream consequences of liver dysfunction, but as candidate transitional features that may refine risk stratification beyond conventional surveillance tools. Despite accumulating evidence, several high priority gaps remain unresolved. First, it is still unclear which microbial or metabolite alterations represent true transitional signals rather than markers of hepatic decompensation or treatment exposure. Second, longitudinal data are insufficient to determine temporal ordering between microbiome shifts, immune remodeling, and tumor initiation. Third, heterogeneity in sampling matrices, analytical platforms, and patient medication exposure limits cross cohort reproducibility. Addressing these issues should be prioritized before microbiome based risk stratification tools can be reliably translated into clinical practice. However, the situational dependence of SCFA effects and confounding factors such as etiology, diet, medication, and complications still significantly affect cross cohort consistency and reproducibility. Therefore, the next step should rely on vertical queues and multi omics integration to construct interpretable combination features and conduct external validation, in order to promote scalable early warning and hierarchical management tools.
